# An annotated dataset of bioacoustic sensing and features of mosquitoes

**DOI:** 10.1038/s41597-020-00725-6

**Published:** 2020-11-11

**Authors:** Dinarte Vasconcelos, Nuno Jardim Nunes, João Gomes

**Affiliations:** 1 ITI/LARSYS, Instituto Superior Técnico, Universidade de Lisboa, Lisboa, Portugal; 2 ISR/LARSYS, Instituto Superior Técnico, Universidade de Lisboa, Lisboa, Portugal

**Keywords:** Ecological epidemiology, Malaria

## Abstract

As vectors of malaria, dengue, zika, and yellow fever, mosquitoes are considered one of the more severe worldwide health hazards. Widespread surveillance of mosquitoes is essential for understanding their complex ecology and behaviour, and also for predicting and formulating effective control strategies against mosquito-borne diseases. One technique involves using bioacoustics to automatically identify different species from their wing-beat sounds during flight. In this dataset, we collect sounds of three species of mosquitoes: *Aedes Aegypti*, *Culex Quinquefasciatus* & *Pipiens*, and *Culiseta*. These species were collected and reproduced in the laboratory of the Natural History Museum of Funchal, in Portugal, by entomologists trained to recognize and classify mosquitoes. For collecting the samples, we used a microcontroller and a mobile phone. The dataset presents audio samples collected with different sampling rates, where 34 audio features characterize each sound file, making it is possible to observe how mosquito populations vary heterogeneously. This dataset provides the basis for feature extraction and classification of flapping-wing flight sounds that could be used to identify different species.

## Abstract (summary)

**Table:** 

Measurement(s)	Sound • audio feature
Technology Type(s)	bioacoustic sensing • audio board • Microphone Device • sensor • mobile phone
Factor Type(s)	species of mosquito • sampling rate
Sample Characteristic - Organism	Aedes aegypti • Culex pipiens x Culex quinquefasciatus • Culiseta
Sample Characteristic - Location	Madeira

Machine-accessible metadata file describing the reported data: 10.6084/m9.figshare.13034597

## Background & Summary

Mosquitoes (Culicidae) are a hazard for millions of people worldwide since they act as vectors for life-threatening pathogens and parasites. Monitoring and predicting mosquito abundance over time, identifying the main determinants of the mosquito population, and assessing control strategies are fundamental activities for implementing timely and effective policies to prevent the spread of vectors. The methods and tools for collecting ecological and behavioral data rely traditionally upon labour-intensive techniques such as sampling of immature stages from breeding sites, systematic search and collection of resting adults, or using sticky ovitraps that detect ovipositing females through their eggs. These methods are slow, often taking weeks to complete, and lack epidemiological sensitivity, in that they are unable to effectively detect host-seeking adults, which is the crucial indicator of risk for disease transmission.

With the widespread availability of low-cost IoT devices and developments in machine learning techniques, bioacoustics is becoming an up-and-coming technique for biodiversity monitoring. Recent work presented bioacoustic solutions based on mobile phones^1^ and low-cost IoT devices^2^ for detecting and classifying spatial and temporal metadata for species identification. The availability of training datasets is crucial for any effective acoustics-based technique to identify and classify wingbeat sounds automatically.

In this study, we present the work that motivated the creation of the bioacoustics dataset and some background on mosquitoes species^3^. We begin by presenting some classical approaches and methods to study mosquitoes, and then discuss the technical approaches for solving similar problems through bioacoustics sensing. Finally, we analyze how the data were evaluated in these preliminary studies, mostly based on the fundamental frequency.

Previous work recorded flight tones of *Aedes Aegypti* mosquitoes (31 males and 28 females) and concluded that the frequency of male wingbeats was higher than females (982 - 721 Hz vs 664 - 514 Hz)^4,5^. These frequency ranges can be influenced by body size, age and temperature^6^. According to a study by Unwin and Corbet in 1984^7^, wingbeat frequency changes proportionally to temperature. Where they presents an analysis taking into account different capture angles in quiet environments. The main disadvantage of sex classification using the fundamental frequency arises when the mosquitoes are in the breeding phase. Here - the frequency of wingbeats of females is more similar to that of males^8–10^ and are thus very hard to distinguish based on that metric alone.

Another interesting approach to study the mosquitoes behavior by Batista *et al*. was to use an inexpensive optical sensor to classify insects throughout the day for three types of mosquitoes automatically. The prediction is based on the period of most significant daily activity, which was mapped from real distributions available in the literature based on the wingbeats frequency^11^, although this sensor has great accuracy, its range is reduced, being necessary to force the mosquito passing through the laser. The classification approach was successfully applied in the wild in^2^ to identify different species automatically.

More recently in^12^, the k-nearest neighbour criterion was used to compute the unknown fundamental frequency distance for each individual mosquito, and identify them using a Bayesian classifier^13^. The underlying dataset is presented in^14^. Others have also used machine learning methods^15,16^ with both temporal and spectral features. One crucial research question for mosquitoes bioacoustic identification is to what extent the changes induced by environmental conditions (location, temperature, time of day, humidity, air density, etc.) impact the pattern recognition algorithms. The availability of public datasets collected in different geographic and environmental conditions is critical to understanding how these issues affect recognition algorithms.

Finally, none of the published datasets includes environmental noise (e.g. wind or ambient noise), which is essential to fully characterize mosquitoes in real world scenarios. Here, we present a dataset with 34 features and different sampling rates that can be used to define a strategy to deal with different environmental factors, to find patterns, or to facilitate efficient audio fingerprinting for each species.

The mosquitoes were caught in the field, and all features have been collected in a laboratory, in the Madeira archipelago, located around 1,000 km from mainland Portugal and around 500 km from the north African coast. The island’s Mediterranean climate exhibits little temperature variation throughout the year.

## Methods

We conducted a laboratory study in the facilities provided by the Natural History Museum of Funchal (Mosquito Lab). Three species of mosquitoes were recorded to determine their dominant frequencies and spectral behaviors. The species used for this collection and study were *A. Aegypti*, *C. Quinquefasciatus* & *Pipiens* and *Culiseta*, which came from a lab colony established from captures collected in Funchal city in 2019.

The mosquitoes were kept in an environmental room simulating natural conditions, with 60 ± 10% relative humidity and temperature of 20–25 °C. Mosquitoes were housed individuals in boxes (25 × 25 × 25 cm) covered with a mesh cap. They were fed with 20% sucrose solution supplemented with 1 g aquarium fish food mixed daily from the brand “Sera Guppy Gran”. The duration of the study was approximately 48 days. All mosquitoes used in these experiments were 7–25 days old. For the recording process, sensors were incorporated into the boxes and the tests conducted on 12–18 specimens for *Aedes Aegypti*, 7–12 specimens for *Culex* and 4 specimens for *Culiseta*. The duration of the extracted sequences ranged from 0 to 300 ms. To generate samples closer to real-world acquisition conditions we added environmental noise in some mosquito samples.

Uncompressed audio of real sound waves was converted to digital format without any further processing. This means that recordings are exact copies of the source audio, recorded in WAV files.

The acoustic sensor uses a low-noise omnidirectional microphone capsule^2^. The microphone converts sound into electrical signals with a specific signal to noise ratio (80 dB), self-noise, and residual noise. All these parameters influence the quality of the acquired sound.

Noise can be a significant problem when acquiring physical signals as voltages. Signal smoothing attempts to capture the essential information in the signal while leaving out the noise. This is done by interpolating the raw signal to estimate the original one^17^.

To collect samples, we used three devices: one of them was our prototype comprising a Teensy 3.2 audio board, microphone and environmental sensor for 44.1 kHz sampling rate. The other two were general-purpose smartphones (Huawei P20 Lite and IPhone 4) used to record samples with a 8 and 48 kHz sampling rate, respectively.

**Fig. 1:**
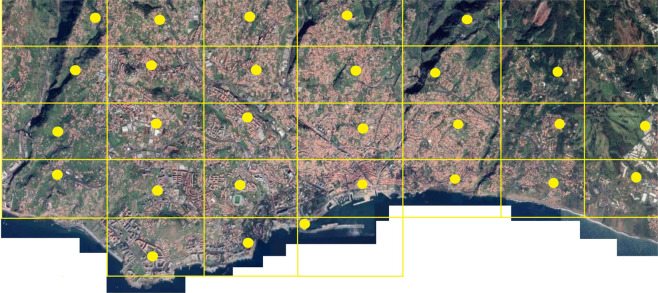
Location and number of traps in the city of Funchal, Madeira, Portugal.

To start a colony for our experience, we installed traps and buckets of water to catch eggs and adult mosquitoes. The female *Aedes* mosquitoes require a blood meal before each egg-laying^18^. The eggs are deposited individually on the inner walls of any container capable of storing water. This work was conducted jointly with the Natural History Museum of Madeira and IASaude (the regional health authority of Madeira islands) as part of a plan to control the spread of mosquitoes in the city of Funchal (Fig. 1).

*A. Aegypti* mosquitoes, lay the most eggs in the velcro tape, while *Culex* and *Culiseta* prefer to lay directly in rafts on still water or in other substances^19^. Traps with a ventilation system were also used to capture adult mosquitoes, especially *Culex* and *Culiseta*.

**Fig. 2:**
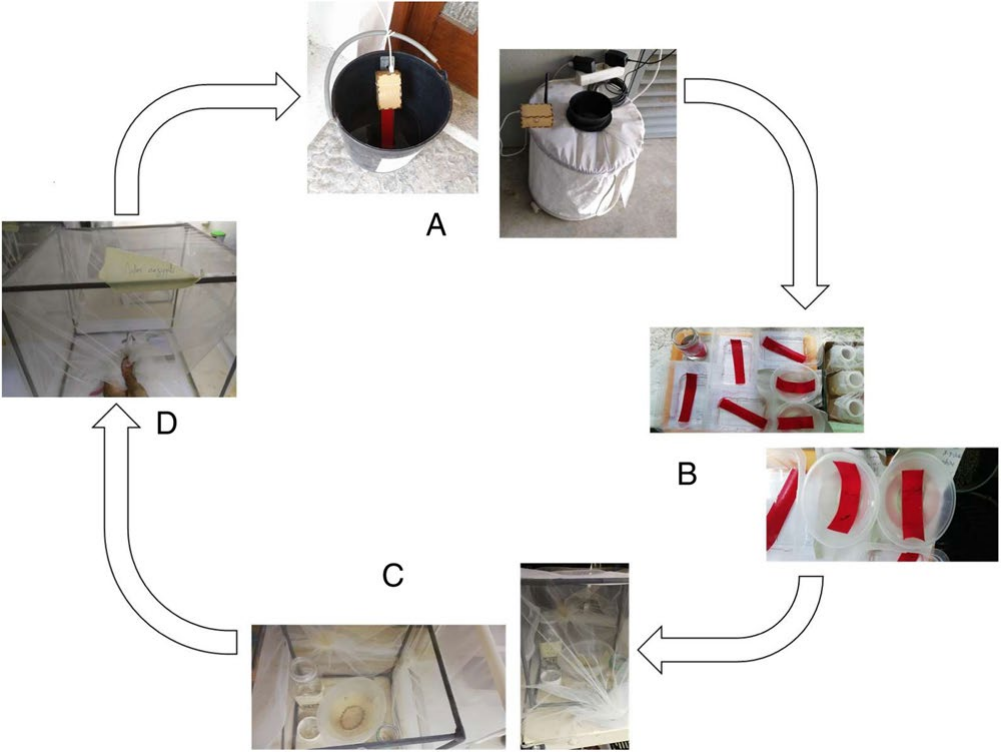
Procedure for collecting audio samples for different species.

Figure 2 shows the procedure from egg collection to mosquito germination, and also the boxes that are used for further acquisition of sound samples. It is noteworthy that after 25–30 days the mosquitoes die due to the conditions imposed in the study.

Step A comprises the gathering of eggs and mosquitoes. The figures show a bucket inside which mosquitoes lay eggs on a velcro tape, and also a trap. These traditional methods allow a fine assessment of the distribution of mosquito populations over time and space (periodically summarized in epidemiological bulletins). In step B the collected eggs are germinated to create a colony. Then, (step C) mosquitoes are placed in boxes and fed with a sugar solution and fish food^20^. Finally, in step D, audio samples are collected by the devices: mobile phones and low-cost IoT. This procedure is repeated when the colony dies after 25 days, starting from step B.

Audio was recorded inside boxes (25 × 25 × 25 cm) where the mosquitoes were located at a maximum distance of 27 cm from the microphone placed in the center of the box. The signal amplitude fluctuates significantly over time as the mosquitoes in free flight approach the microphone or move away.

Continuous recordings were then split into 300 millisecond (ms) snippets. Since mosquitoes have a very short flight, it was necessary to apply a slight stimulus on the wall of the boxes (covered by a net) to force them to fly.

To analyze each mosquito recording, 34 features were extracted taking into account several parameters of the signal belonging to three different domains: time (1–3), frequency (4–8, 22–34) and cepstrum (9–21), analyzed below in the Technical Validation section^21,22^.

These features are often used for speech signal classification, but are useful when handling non-speech signals as well. They enable a comprehensive analysis of the mosquito sounds in terms of amplitude, energy, zero crossing rate, power, frequency variation in the audio file, tonality, loudness, etc. The features are included in the dataset^23^ and their computation is demonstrated in the Code Availability section.

## Data Records

**Fig. 3:**
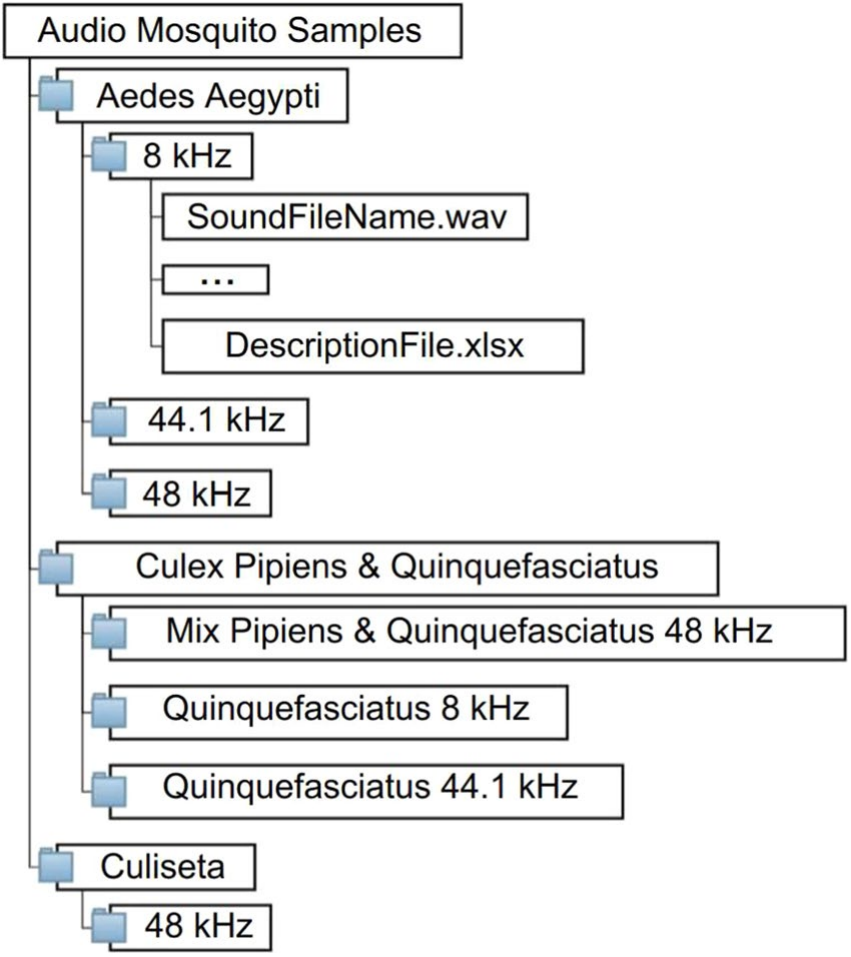
File organization of the dataset.

The files are organized by folders, where the main folder is the name of the mosquito species, with sub-folders organized by sample rate. Inside the sub-folders, and for different sampling rates (8, 44.1 and 48 kHz) of each species, are the associated 300 ms sound snippets and a CSV file identifying each snippet file and its 34 features mentioned above. The diagram presented in Fig. 3 exemplifies how the data are distributed and organized.

The diagram provides an overview of the data files contained in each folder and their formats. The dataset is in the figshare repository, where it is possible to find the insect statistics, bioacoustic recordings and the wing-beat frequency^23^. Table 1, indicates the number of samples for each species.

**Table 1:** Number of features for each species and corresponding sampling rate!.

	Aedes Aegypti	Mix Pipiens & Quinquefasciatus	Culex Quinquefasciatus	Culiseta
**8 kHz**	1 697	—	867	—
**44.1 kHz**	89	—	17	—
**48 kHz**	90	156	—	72

## Technical Validation

**Fig. 4:**
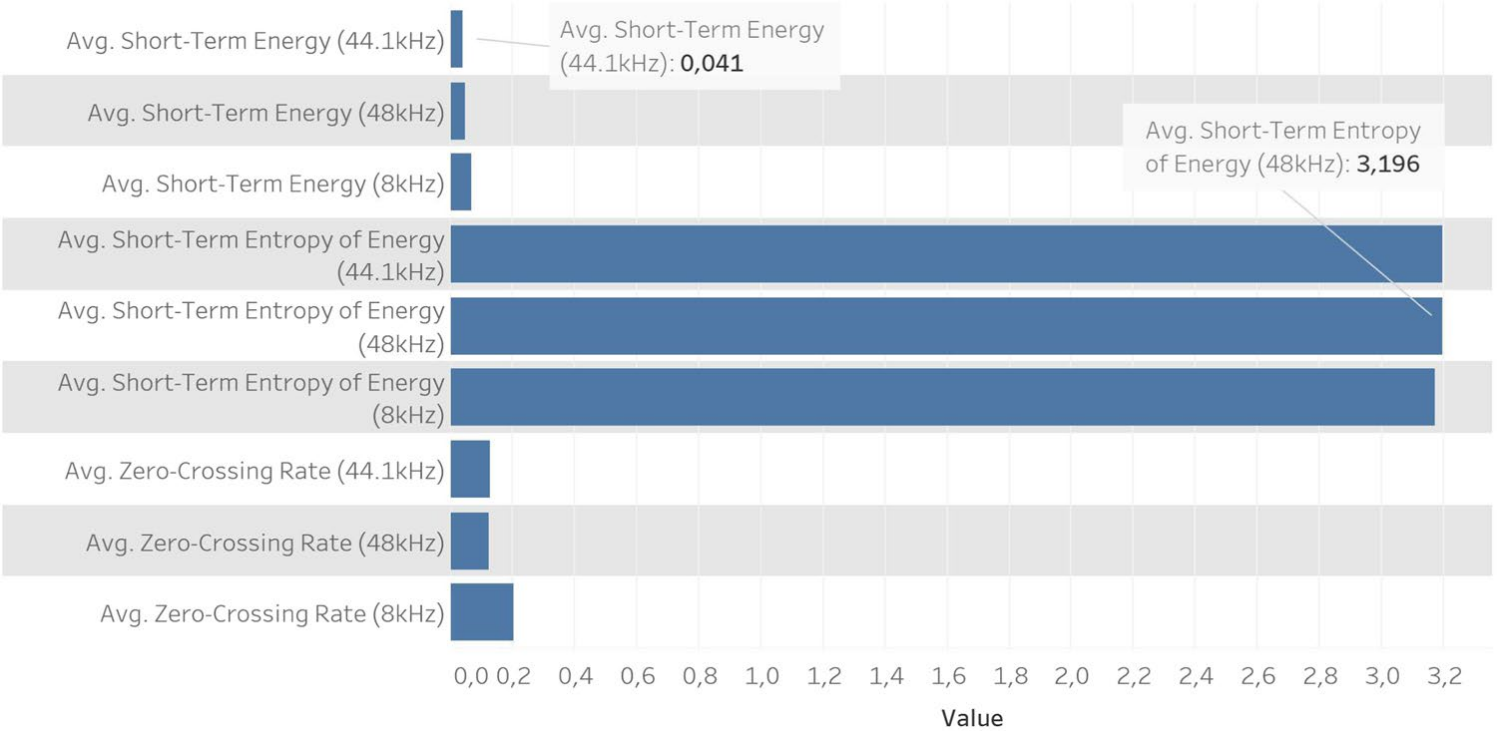
Comparison between time-domain features for 8 kHz, 44.1 kHz & 48 Khz sampling rates.

In this section, we present a structural analysis to support the interpretation of the dataset. This analysis is conducted through 4 fields, based on metrics derived in the time-domain, frequency-domain, cepstral-domain/MFCCs, and Chroma Vector. Numerical results are given for *Aedes Aegypti* only, as the statistics are similar for the remaining species. However, each species exhibits a distinctive pattern in terms of frequency and spectrum. Figure 4 shows the average for the three time-domain features, all having a weak dependence on the sampling rate. The zero-crossing rate represents the number of sign-changes of the signal for one complete frame; the energy is the sum of squares of the signal normalized by its length and the Entropy of Energy can be interpreted as a measure of abrupt changes. Using a sampling rate of 8 kHz we have a higher average for Short-Term Energy and Zero-Crossing Rate, however for the Short-Term Entropy of Energy the highest value is attained at 48 kHz.

**Fig. 5:**
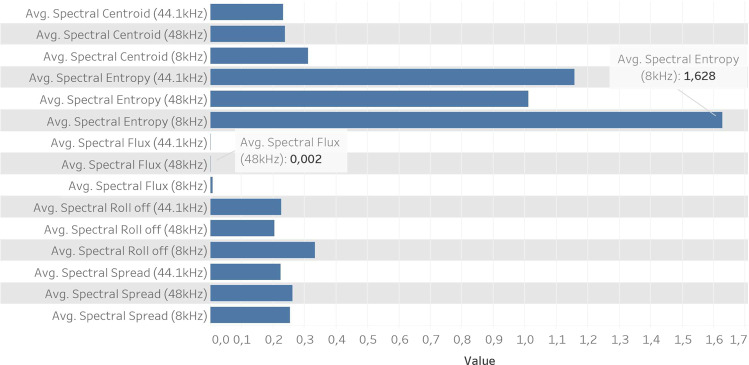
Comparison between 5 spectral features for 8 kHz, 44.1 kHz & 48 kHz sampling rates.

For the second set of features, computed in the frequency domain, we have a different behavior, which is strongly influenced by the sampling rate. The first feature is the center of gravity, which is related to the impression of brightness of the sound, and the second feature captures the peakiness of a distribution (normalised spectral energy for a set of sub-frames). The Roll Off feature quantifies the frequency below which 90% of the magnitude is concentrated. The last feature quantifies the average spread of the spectrum relative to its centroid. A high spectral spread represents a noisy sound, making it challenging to extract useful information. Figure 5 outlines the spectral features, where spectral entropy and Roll Off present a higher average for 8 kHz sampling rate. The average of Spectral Flux attains the lowest value of these features and represents the square difference between the normalized magnitudes of the spectrum for two consecutive frames.

**Fig. 6:**
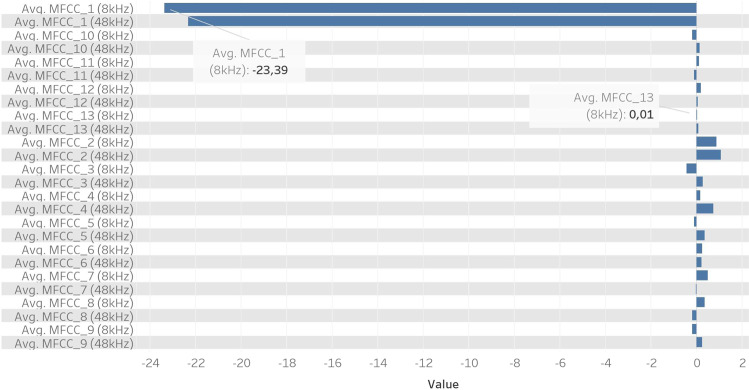
Comparison between 13 MFCCs features for 8 kHz & 48 kHz sampling rates.

Figure 6 presents the analyzed Mel Frequency Cepstral Coefficients (MFCC) over a set of frequency bands that are distributed non-linearly according to the Mel scale. Note the strong general dependence of these average coefficients on the sampling frequency, with several algebraic sign reversals between 8 kHz and 48 kHz. Results for 44.1 kHz are omitted in the interest of space, as they are almost identical to those for 48 kHz. The highest absolute value is attained for MFCC_1 (8 kHz) and the lowest one for MFCC_13 (8 kHz).

**Fig. 7:**
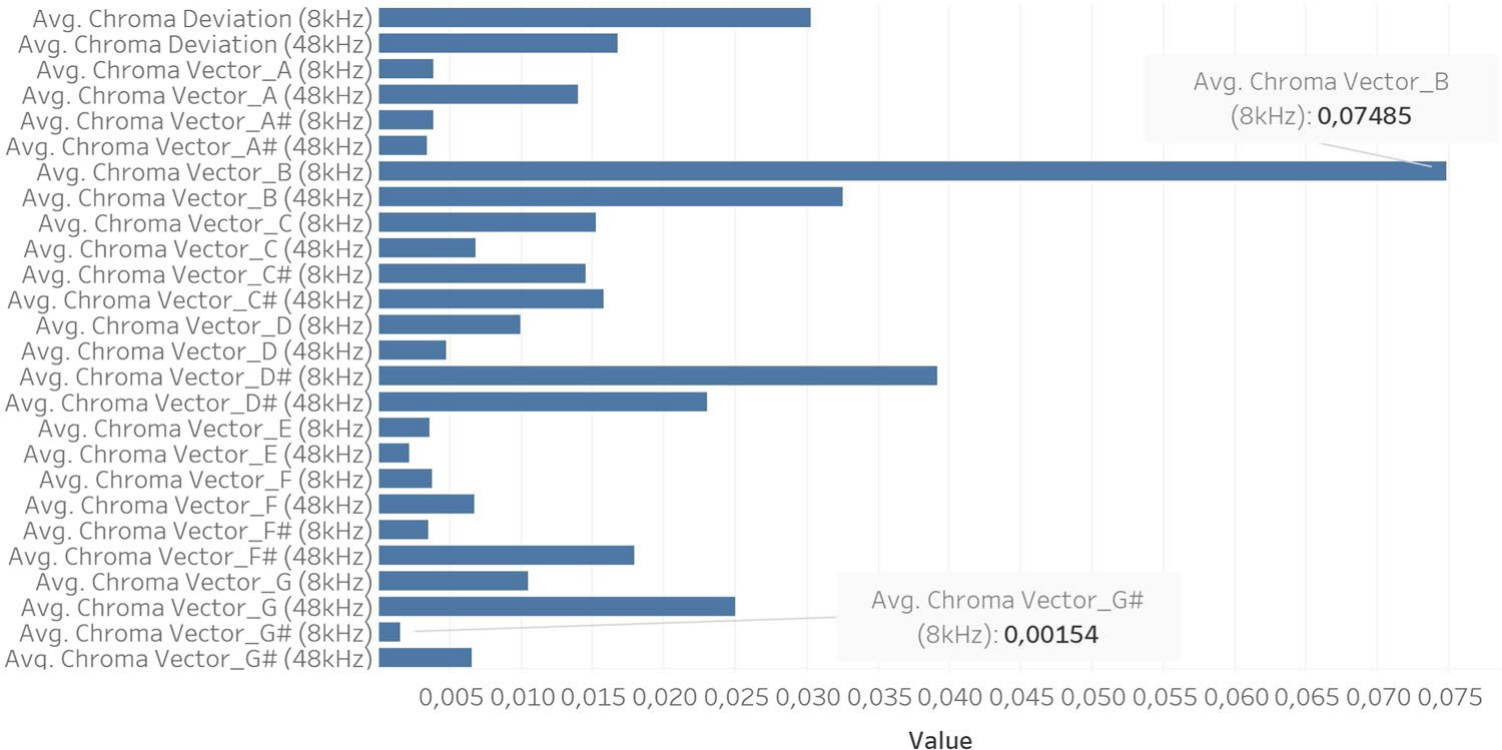
Comparison between 12 Chroma Vectors and Chroma Deviation features for 8 kHz & 48 kHz sampling rates.

The last group of features analyzed are the Chroma Vectors. They have 12 elements that represent the harmonic and melodic content of the sound. These have the most discrepant values for different sampling rates, as shown in Fig. 7. Chroma Vectors A, C#, F, F#, G and G# attain the highest values for a sampling rate of 48 kHz. The most distinctive feature is Chroma Vector B at 8 kHz, with a value of 0.07485. The standard deviations of the Chroma vectors for 8 kHz are almost doubled when compared to those for 48 kHz.

## Usage Notes

The dataset can be publicly accessed through a free and open-source platform for broad dissemination and use^23^, where the data are organized in folders as described in the Data Records section.

Researchers can reuse the data downloading it as a zip archive. In addition to the audio files, a CSV database is associated with it, to make it possible to check the characteristics of each sample for each type of mosquito using temporal, frequency, spectral and MCFFs features.

To reproduce the representative features computed from WAV files, readers can use and replicate the algorithm listed in the code availability section. For that they need to install the open-source Anaconda Distribution platform with python 2.7 or 3 version and the following packages: numpy, matplotlib, scipy, sklearn, hmmlearn, simplejson, eyed3, pydub and glob.

Box 1: Algorithm for supervised segmentation using Ipython.

1. from pydub import AudioSegment
  2:
2. newAudio = AudioSegment.from_wav(“Uninterrupted_Filename.wav”)
3. ▷ ms
4. time_slip = 300
5. all_time = len(newAudio)
6. number_of_files = len(newAudio)/time_slip
  8:
7. number_file = 0
8. for i in range(number_of_files + 1):
9. t1 = i*time_slip
10. t2 = (i + 1)*time_slip
11. new Audio = newAudio[t1:t2]
  14:
12.            ▷ Exports to a wav file in the current path
13. newAudio.export(“filename_“ + str(number_file) + “.wav” format = “wav”)
14. number_file = number_file + 1
  18:

Box 2: Algorithm to reproduce the features and csv file using Ipython.

1. import numpy, glob, csv
2. from pyAudioAnalysis import audioFeatureExtraction as aF
  3:
3. ▷ Extract the features and species name
  5:
4. [features, classNames, fileNames] = aF.dirsWavFeatureExtraction([’Folders of each species’], 0.3, 0.15, aT.shortTermWindow, aT.shortTermStep, computeBEAT = False)
5. ▷ Deletion of files that have NaN features
6. for f in features:
7. fTemp = []
8. for i in range(f.shape[0]):
9. temp = f[i,:]
10. if (not numpy.isnan(temp).any()) and (not numpy.isinf(temp).any()):
11. fTemp.append(temp.tolist())
12. else:
13. print “NaN Found! Feature vector not used for training”
14. features2.append(numpy.array(fTemp))
15. features = features2
  18:
16. print’Number of species:’ + str(len(features))
17. print’Number of files of each species:’ + str(len(features[“id of each species”]))
18. ▷ Features
19. namFeatures = [’zero crossing rate’,’short-term energy’,’short-term entropy of energy’,’spectral centroid’,’spectral spread’,’spectral entropy’,’spectral flux’,’spectral rolloff’,’MFCCs_1’,’MFCCs_2’,’MFCCs_3’,’MFCCs_4’,’MFCCs_5’,’MFCCs_6’,’MFCCs_7’,’MFCCs_8’,’MFCCs_9’,’MFCCs_10’,’MFCCs_11’,’MFCCs_12’,’MFCCs_13’,’Chroma Vector_A’,’Chroma Vector_A#’,’Chroma Vector_B’,’Chroma Vector_C’,’Chroma Vector_C#’,’Chroma Vector_D’,’Chroma Vector_D#’,’Chroma Vector_E’,’Chroma Vector_F’,’Chroma Vector_F#’,’Chroma Vector_G’,’Chroma Vector_G#’,’Chroma Deviation’]
20. ▷ Print all the wave files
21. print(glob.glob(“Folders of each species/*.wav”))
22.                          ▷ Creating csv file
23. with open(’Specie.csv’, mode = ’w’) as csv_file:
24. writer = csv.DictWriter(csv_file, fieldnames = namFeatures)
25. writer.writeheader()
26. csv_writer = csv.writer(csv_file, delimiter = ’,’, quotechar = ’“’, quoting = csv.QUOTE_MINIMAL)
27. for i in range(34):
28.             csv_writer.writerow(features[0][0:len(features[0]),i])

## Data Availability

Box 1 describes the algorithm to segment the audio (WAV file) into snippets of 300 ms. Supervised segmentation is a critical process for most of the audio analysis applications, its purpose being to split an audio stream into homogeneous segments. We used the pyaudioanalysis library to generate and extract 34 features, represented in Box 2. This is an open python library that provides audio related functionalities such as: audio features, visualization, classification and segmentation^22^. Box 2 shows the function to create, extract and manipulate the 34 features. Both algorithms can be found in the Github repository^24^. We used the library audio feature extraction from pyaudioanalysis using the method *dirWavFeatureExtraction()* to extract the short-term feature sequences of WAV files (one audio signal per specimen), using a sliding window with a time overlap of 50% (frame size of 300 ms and frame step of 150 ms). The resulting 34-element feature vector for each audio file is extracted by mid-term averaging the short-term features.
